# Exocytosis Proteins: Typical and Atypical Mechanisms of Action in Skeletal Muscle

**DOI:** 10.3389/fendo.2022.915509

**Published:** 2022-06-14

**Authors:** Jinhee Hwang, Debbie C. Thurmond

**Affiliations:** Department of Molecular and Cellular Endocrinology, Arthur Riggs Diabetes and Metabolism Research Institute, Beckman Research Institute at City of Hope, Duarte, CA, United States

**Keywords:** SNARE protein, glucose transporter-4 (GLUT4), glucose uptake, skeletal muscle, insulin resistance, type 2 diabetes

## Abstract

Insulin-stimulated glucose uptake in skeletal muscle is of fundamental importance to prevent postprandial hyperglycemia, and long-term deficits in insulin-stimulated glucose uptake underlie insulin resistance and type 2 diabetes. Skeletal muscle is responsible for ~80% of the peripheral glucose uptake from circulation *via* the insulin-responsive glucose transporter GLUT4. GLUT4 is mainly sequestered in intracellular GLUT4 storage vesicles in the basal state. In response to insulin, the GLUT4 storage vesicles rapidly translocate to the plasma membrane, where they undergo vesicle docking, priming, and fusion *via* the high-affinity interactions among the soluble N-ethylmaleimide sensitive factor attachment protein receptor (SNARE) exocytosis proteins and their regulators. Numerous studies have elucidated that GLUT4 translocation is defective in insulin resistance and type 2 diabetes. Emerging evidence also links defects in several SNAREs and SNARE regulatory proteins to insulin resistance and type 2 diabetes in rodents and humans. Therefore, we highlight the latest research on the role of SNAREs and their regulatory proteins in insulin-stimulated GLUT4 translocation in skeletal muscle. Subsequently, we discuss the novel emerging role of SNARE proteins as interaction partners in pathways not typically thought to involve SNAREs and how these atypical functions reveal novel therapeutic targets for combating peripheral insulin resistance and diabetes.

## 1 Introduction

The global diabetes prevalence has increased over the last 50 years, and due to diabetic complications and a lack of therapies, diabetes has become one of the leading causes of mortality and morbidity. According to the International Diabetes Federation evaluation in 2021, approximately 537 million people (10.5%) worldwide have diabetes (1). Diabetes prevalence is expected to continue increasing, with a 48% increase in diabetes cases by 2045 (1).

Diabetes is typically divided into type 1 (T1D) and type 2 (T2D), with 5-10% of diagnoses being T1D and 90-95% of cases being T2D. T1D is characterized by dysfunction and demise/loss of insulin-producing pancreatic β-cells, leading to elevated blood glucose levels; T2D is characterized by a progressive reduction in peripheral insulin sensitivity (i.e., insulin resistance), known as prediabetes, which elevates blood glucose levels and eventually disrupts β-cell function and cell viability as the disease progresses to T2D (2). Insulin resistance manifests as an inability of insulin to facilitate glucose uptake and utilization in the body. As skeletal muscle is responsible for ~80% of postprandial glucose uptake (3), insulin resistance in skeletal muscle severely impedes glucose disposal in patients with prediabetes and T2D (4). Importantly, the crucial rate-limiting step in glucose uptake into skeletal muscle is the translocation of the insulin-stimulated glucose transporter type 4 (GLUT4) protein to the cell surface (5). Upon insulin stimulation, GLUT4 storage vesicles (GSVs) translocate from internal cellular compartments out to the cell surface, where they undergo docking and fusion by the formation of heteromeric soluble N-ethylmaleimide sensitive factor attachment protein receptor (SNARE) protein complexes. SNARE core complexes include one GSV-associated SNARE protein (VAMP2) and two plasma membrane (PM)-localized SNARE proteins (STX4 and SNAP23) (6). It has been reported that this GLUT4 vesicle translocation process is much reduced in individuals with insulin resistance than in healthy individuals (7).

Numerous reports reveal that peripheral insulin resistance and T2D susceptibility are correlated with deficiencies in SNAREs and their regulators (8–21). Furthermore, deficits in SNARE and other exocytosis proteins have been linked to other diseases such as cancer, neutrophil-mediated disease, bleeding, and neuronal disorders (22–31), indicating that these proteins may be meaningful therapeutic targets across a wide range of diseases. In this review, we highlight the latest advances in the molecular and structural characterization of SNAREs and their regulatory proteins. We also pay particular attention to the unexpected newly-discovered protein:protein interactions considered uncharacteristic for SNARE proteins, which expand prior thinking of their physiological roles beyond that of exocytosis proteins. We also discuss how targeting the typical and atypical SNARE functions might facilitate reversal of peripheral insulin resistance and combat T2D.

### 1.1 Introduction to GLUT4 Vesicle Translocation and Insulin Signaling

GSV translocation to, and deposition of the GLUT4 protein into, the PM requires a combination of insulin signaling and vesicle trafficking pathways. The journey initiates once insulin binds to the extracellular α-subunit of the tetrameric insulin receptor, which spans the PM of skeletal muscle cells/fibers (32), and triggers canonical insulin signaling cascades. Intracellular auto-tyrosine-phosphorylation of the insulin receptor β-subunit and subsequent phosphorylation of the insulin receptor substrate-1 (IRS-1) ensue, enabling phosphatidylinositol-3-kinase (PI3K) activation. Of note, PI3K activation bifurcates into two parallel intracellular insulin signaling arms, both of which promote GSV translocation and GLUT4 deposition at the cell surface of muscle cells: 1) activation of the serine/threonine kinase AKT in the canonical pathway, and, 2) activation of the Rho-family GTPase Rac1 in the noncanonical pathway. Most studies using pharmacological inhibition or gain- and loss-of-function studies have revealed that the AKT and Rac1 pathway arms are independent. Neither AKT inhibition nor AKT dominant-negative mutants affect insulin-stimulated Rac1 activation or consequent actin remodeling (33), and Rac1 knockout in mice does not abolish AKT phosphorylation by insulin in skeletal muscle (34). Moreover, perturbation of either AKT or Rac1 signaling significantly diminishes insulin-stimulated glucose uptake, and simultaneous inhibition of both fully blocks insulin-stimulated glucose uptake (34–36), suggesting that both signaling arms are required for GSV-mediated glucose uptake.

#### 1.1.1 Canonical Insulin Signaling Pathway

PI3K phosphorylates phosphatidylinositol 4,5-biphoshate (PIP_2_) to phosphatidylinositol 3,4,5-triphosphate (PIP_3_) at the PM, which recruits AKT. AKT is activated by phosphorylation at Thr308 by phosphoinositide-dependent kinase-1 (PDPK1) and phosphorylation at Ser473 by mTORC2. AKT has three isoforms (AKT1, AKT2, and AKT3) in mammalian cells. Of them, AKT1 and ATK2 are highly expressed in skeletal muscle, and AKT2 is particularly known as a key player regulating glucose transport into skeletal muscle (37–39). Activated AKT phosphorylates the Rab GTPase-activating protein (GAP) such as AS160/TBC1D4 and TBC1D1; phosphorylated AS160/TBC1D4 at Thr642 and Thr596 and TBC1D1 at Thr590 then lowers the TBC1D4 and TBC1D1 GAP-activity towards Rab GTPases on the GSV, leading to GSV mobilization and/or fusion. Under basal conditions, the unphosphorylated active form of AS160/TBC1D4 promotes hydrolysis of GTP to GDP by Rab proteins on the GSV. Thus, more GDP-bound Rab forms, and GSV translocation to the PM is stalled. In contrast, activated AKT phosphorylates AS160/TBC1D4 and TBC1D1, thereby inactivating its GAP activity. Thus, inhibition of GAP alters the equilibrium of GDP-bound Rab to GTP-bound Rab, allowing translocation of GSVs to the PM.

#### 1.1.2 Noncanonical Insulin Signaling Pathway

In parallel to Akt activation, PI3K produces PIP_3_ at the PM, which recruits guanine nucleotide exchange factors (GEFs) and activates Rho-family GTPases, especially Rac1. This GTPase activation promotes actin remodeling, which moves GSVs out to the PM. In brief, Rac1 activates the downstream effector protein kinase p21-Activated kinase 1 (PAK1). In skeletal muscle cells, pharmacological inhibition of PAK1 diminished insulin-stimulated GLUT4 translocation and glucose uptake by blunting insulin-stimulated cortical actin remodeling and cofilin activation (40). Further, in muscle cells stimulated with insulin, activated PAK1 triggers activation of its effector protein p41ARC, a subunit of the Arp2/3 complex, and increases the association of p41ARC with N-WASP. N-WASP was coordinately connected to cortactin-mediated actin polymerization and GLUT4 vesicle translocation (41). Global and skeletal-specific PAK1 knockout mice displayed insulin resistance and reduced insulin-stimulated GLUT4 translocation (42, 43); this observation is reminiscent of GLUT4 whole-body knockout (44). Skeletal-specific PAK1 overexpressing mice enhance glucose tolerance, requiring PAK1 for glucose homeostasis (43). In contrast, another study of the global PAK1 knockout mice reported normal insulin-stimulated glucose uptake in skeletal muscle *ex vivo* (45). However, the conclusions drawn from the older non-inducible global PAK1 knockout model are complicated by the potential for differential impact upon embryonic development, and the potential for differential compensatory mechanisms. The skeletal muscle-specific deletion of PAK1, deleted inducibly and only in adult mice, circumvents these caveats. Nevertheless, additional studies using new genetic mouse models will be needed to validate factors identified in this new downstream signaling cascade as required for insulin-stimulated GLUT4 vesicle translocation and glucose uptake in primary skeletal muscle *ex vivo* and *in vivo*.

### 1.2 Role of SNARE Proteins in GLUT4 Translocation

After GLUT4 vesicles arrive at the PM in response to insulin, they dock and fuse with the PM *via* SNARE protein interactions (46, 47), ultimately inserting the GLUT4 protein into the PM. Functionally, two distinct types of SNAREs control exocytosis: v-SNAREs, which localize to the transport vesicle membrane (e.g., GSV), and t-SNARES, which localize to the target membrane (e.g., PM). The t-SNAREs that facilitate GSV exocytosis are syntaxin 4 (STX4) and synaptosomal-associated protein 23 (SNAP23), and the v-SNARE is vesicle-associated membrane protein 2 (VAMP2) (12, 48, 49). The t-SNAREs are present in monomeric or binary conformations on the PM, and VAMP2 on the GSV is monomeric. The fusion event is driven through the formation of a ternary trans-SNARE core complex assembly that combines one v-SNARE with two cognate t-SNAREs in a heterotrimeric 1:1:1 ratio (50, 51). This ternary SNARE assembly is energetically favorable, so there is sufficient energy to catalyze membrane fusion by forcing the two membranes closely together (52). Following fusion, the SNARE complex becomes the cis-SNARE complex in a contiguous PM structure, with the GLUT4 protein intercalated. SNARE complexes are notoriously heat- and SDS-resistant (53). After fusion, cis-SNARE complexes are dissembled by binding to the N-ethylmaleimide-sensitive factor (NSF) and α-soluble NSF attachment proteins (α-SNAPs) for recycling of the proteins (54).

The formation of the SNARE core complex is further regulated through SNARE regulatory proteins such as STX4-interacting protein (synip) (55), the mammalian homolog of UNC-18 (Munc18c) (17, 56), tomosyn-1 (57), and double C2-like domain-containing protein B (DOC2B) (20, 58). Synip, tomosyn-1, and Munc18c directly bind with STX4 (55, 57, 59). Several studies have demonstrated that overexpressing tomosyn-1 or Munc18c precludes GSV docking and SNARE-dependent fusion (60, 61). In contrast, DOC2B is a positive regulator of SNARE-dependent GSV fusion in skeletal muscle, coordinating the activation/opening of STX4 to promote SNARE complex assembly (20). In addition, complexin-2 regulates the stability of SNARE complexes in skeletal muscle cells (62). This review will discuss the most recent detailed mechanistic findings related to SNARE-mediated GLUT4 vesicle docking and fusion in the following sections.

## 2 Structure of SNARE Proteins Relevant to Membrane Fusion

SNARE proteins were first identified as exocytosis proteins that regulate membrane fusion in the late 1980s. The SNARE proteins consist of a large protein superfamily with approximately 36 members in mammals (63, 64). All SNAREs have a core structure containing a common SNARE motif, a conserved stretch containing 60-70 amino acids arranged in heptad repeats, and structurally divergent N-and C-termini (64). The v-SNARE VAMP subfamilies contain either a long domain or a short unstructured peptide at the N-terminus, a conserved SNARE motif, short linkers, and a C-terminal transmembrane domain (TMD) (63, 65). The t-SNARE STX subfamilies consist of an autonomously folded three-helix domain called the H_abc_-domain at the N-terminus, a conserved SNARE motif, short linkers, and a C-terminal TMD (63). The t-SNARE SNAP subfamilies do not have a C-terminal TMD but contain instead two different SNARE motifs (63) joined by a linker that is palmitoylated at cysteine residues (66) or is prenylated (67).

### 2.1 The Role of SNARE Motifs in SNARE Complex Assembly

SNARE motifs primarily mediate heterotrimeric SNARE complex formation. Individual SNARE motifs have a secondary structure, which consists of heptapeptide repeats in an α-helical configuration, with one side of the helix containing all the hydrophobic residues and the other side containing hydrophilic residues (68, 69). For assembly of the heterotrimeric SNARE core complex, the SNARE motifs spontaneously form elongated coiled-coils of the intertwined parallel four α-helical trans-bundle, with the STX and VAMP proteins each contributing one helix and the SNAP protein contributing two helices. Inside the SNARE bundle (e.g., STX1:SNAP25:VAMP2), the four helices form 16 layers (numbered from -7 to +8) of interacting surfaces consisting of amino acid side chains that are mostly hydrophobic and are organized parallel to the axis of the four-helical bundle (70). However, the exception is the central ‘0’ layer. The ‘0’ layer is hydrophilic, consisting of three highly conserved glutamine (Q) residues, one derived from STX and two derived from SNAP, and one highly conserved arginine (R) residue derived from VAMP.

Accordingly, SNAREs are structurally classified into Q-SNAREs and R-SNAREs based on the presence of either a Q or R at this position (70, 71). Q-SNAREs are further classified into Qa (STX subfamily), Qb (subfamily containing the N-terminal motif of SNAP), and Qc (subfamily containing the C-terminal motif of SNAP) SNAREs (71, 72). While it is presumed that STX4:SNAP23:VAMP2 complexes overseeing GSV exocytosis are structurally arranged in the manner described for STX1:SNAP25:VAMP2 complexes above, crystallographic structures have yet to be solved for these isoforms. Recently, isothermal calorimetry and surface plasmon resonance methodologies have provided new insights into STX4-based SNARE complex structure and function. The similarities and differences, which are largely related to the STX4 N-terminus, will be discussed in the next section.

### 2.2 The Role of the SNARE N-Terminus in SNARE Assembly

**Figure 1:**
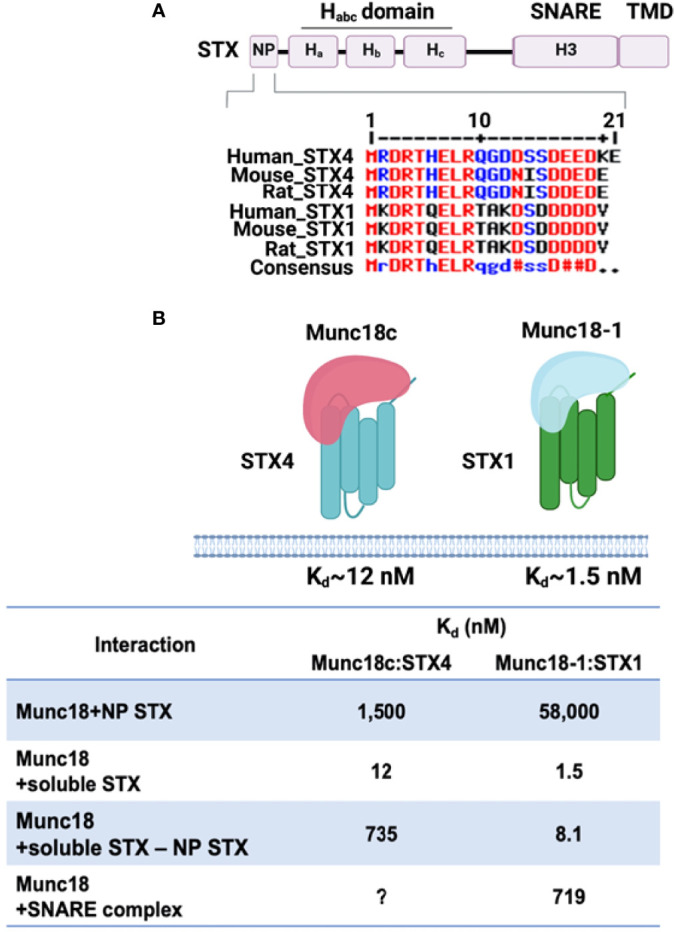
Structure of STX proteins and STX-Munc18 affinity. **(A)** Schematic diagram of the general domain structure of STX (STX1a and STX4). These STXs have a short N-terminal peptide (NP), three-helix bundles (Ha, Hb, and Hc), a SNARE motif, and a transmembrane domain (TMD). The NP sequence homology for STX4 and STX1 is shown. Multiple sequence alignment of human, mouse, and rat STX4 and STX1using Multiple sequence alignment with hierarchical clustering tool (Multalin) was used: Red, high consensus; Blue, low consensus; Black, neutral consensus; Uppercase letter in the consensus line, a residue that is highly conserved; lowercase letter in the consensus line, a residue that is weakly conserved; # in the consensus line, any residue of NDQEBZ; Dot in the consensus line, a position with no conserved. **(B)** The binding affinities of the STX1:Munc18-1 and STX4:Munc18c interactions are indicated. The question mark (?) denotes the absence of data testing the interaction of Munc18c with the STX4-based SNARE complex. Created with BioRender.com and licensed for publication.

In contrast to the conserved SNARE motifs, the N-terminal domains of SNAREs are diverse. The three-helical H_abc_ domain of the Qa-SNARES (STX subfamily) possesses distinct biological functions that depend on the STX subfamilies. For example, the H_abc_ domain of STX1, STX4, and STX7 folds into a three-helix bundle with a left-handed twist and interacts with the C-terminal SNARE motif in an autoinhibited manner, generating a closed conformation, thus preventing the SNARE motif from forming a SNARE complex (73–75). In contrast, the three-helical domains of other STXs, such as STX5 and STX16, do not bind to the C-terminal SNARE motif and exhibit an open conformation (76, 77). To drive membrane fusion, the closed conformation of STX is opened before and/or during the assembly of the heterotrimeric SNARE core complex. Hence, the short peptide region of the N-terminal domain provides a binding site for other proteins, such as Munc18 proteins. In the “closed” conformation, the arch-shaped Munc18 protein clasps the STX bundle (three H_abc_ helices and the SNARE motif). In the “open” conformation, the Munc18 protein has been observed to dissociate, when assessed in primary muscle lysates (20). *In vitro* assays of the Munc18 proteins with their cognate STX isoform partners showed that binding could occur *via* the far N-terminal peptide (NP), upstream of the three STX H_abc_ helices (78) (**Figure 1**). However, these are low affinity interactions: the binding of Munc18c to the STX4 NP K_d_ ~1,500 nM, and binding affinity of Munc18-1 for STX1 NP K_d_ ~58,000 nM, as determined using isothermal titration calorimetry (ITC) (79). This is in contrast to the high binding affinity of Munc18c for the nearly full-length STX4 (soluble STX4, lacks the TMD), K_d_ ~12 nM (75), Munc18-1:soluble STX1, ~1.5 nM. Munc18c binding affinity to soluble STX4 lacking the NP changes dramatically, K_d_ ~735 nM. Interestingly, Munc18-1 binding affinity to soluble STX1 lacking the NP changes less dramatically (K_d_ ~8.1 nM) (75, 80). This new ITC-based data on STX4:Munc18c distinguishes the dynamics of Munc18c:STX4 from that of Munc18-1:STX1 conformations, localizing the difference to the N-terminal peptide, and reconciles long-standing confusion that stemmed from the assumption that these complexes must be fully homologous in structure and function (81). What remains to be completed however are the affinities of Munc18c for the STX4-based SNARE complex. Munc18-1 binding to the STX1-based SNARE complex K_d_ ~ 719 nM, a relatively low K_d_; although the physiologic relevance of the apparent low affinity binding remains to be determined. Indeed, the physiological target of SM proteins in promoting fusion is the membrane anchored trans-SNARE complex, which cannot be recapitulated using truncated or detergent-solubilized proteins. It remains to be determined in both cases the impact of deleting the NP from the STX proteins in the context of the SNARE core complex and binding affinity changes for their cognate Munc18 partners. Nevertheless, the NP data has prompted the skeletal muscle field to screen for factors associated with the full STX4 N-terminus, including the H_abc_ domain linked to the NP, that are selective for GSV translocation and fusion. This is particularly relevant to the skeletal muscle field, since STX4 is the sole STX family member identified in the process of GLUT4 exocytosis. No redundant PM-localized alternate STX isoforms are known to exist.

### 2.3 Roles of the SNARE C-Terminal Transmembrane Domains in Membrane Fusion

Extensive investigations have revealed that the length of the SNARE TMD is important for membrane fusion. The length of the STX TMD varies - the TMD of PM-localized STX proteins (~25 amino acids) is longer than that of other STX family members localized to cis-Golgi (~17 amino acids). It was shown in 3T3-L1 adipocytes that the longer TMD confers STX localization to the PM (82). Furthermore, deletion of the C-terminal half of the v-SNARE Snc2p TMD in yeast blocks SNARE-mediated membrane fusion (83), and shortened TMDs in SNARE-mimics completely blocked fusion (84). Similarly, deletion or insertion mutants in the C-terminal half of the VAMP2 TMD cause deficits in SNARE-mediated neurosecretion in PC12 cells (85).

TMD conformational flexibility is also important for fusion activity. Replacing the VAMP2 TMD with helix-destabilizing β-branched residues (Ile/Val) in chromaffin cells accelerates fusion pore expansion, whereases replacing the VAMP2 TMD with the helix-stabilizing Leu residue strongly impairs exocytosis and slow fusion pore dilation (86). It is thought that poly-Ile and poly-Val substitutions increase TMD flexibility while poly-Leu substitution decreases this flexibility (87). Moreover, the SNARE TMD plays a critical role in promoting the transition from hemifusion to full fusion, as the formation of the fusion pore is initiated by the movement of the VAMP2 TMD uncharged C-terminus into the membrane interior (88). In line with this, several studies have shown that the addition of charged residues to the uncharged C-terminus of the VAMP2 TMD inhibits pore expansion, suggesting that the conformational flexibility of uncharged residues in the TMD is essential for fusion pore formation (84, 89, 90).

## 3 The Physiologic and Mechanistic Roles of SNAREs in GLUT4 Vesicle Translocation and Glucose Uptake in Skeletal Muscle

### 3.1 t-SNAREs

Eleven t-SNAREs have been identified in skeletal muscle and adipocytes. Of these, only the t-SNAREs STX4 and SNAP23 are known to be required for insulin-stimulated GLUT4 vesicle fusion at the PM in skeletal muscle (47).

#### 3.1.1 STX4

Global whole body STX4 heterozygous (-/+) knockout mice are viable but manifest impaired insulin-stimulated GLUT4 translocation and decreased glucose uptake in skeletal muscle (21), emphasizing an important role for STX4 in glucose homeostasis. STX4 homozygous (-/-) knockout mice die early (before embryonic day 7.5), due to decreased fusion of GLUT8-containing vesicles with the PM in the embryos, which causes apoptosis and death (21, 91). Consistent with the results from STX4 (-/+) mice, mice with global overexpression of STX4 show increased insulin-stimulated glucose uptake and GLUT4 deposition in the sarcolemma and t-tubule membranes of skeletal muscle (92). Indeed, these global STX4-overexpressing mice show extraordinary peripheral insulin sensitivity, which increases healthspan and confers a ~35% increase in lifespan, compared to littermate control mice (16). Unlike other highly specialized cell types, such as the insulin-producing β-cell, skeletal muscle GSV exocytosis relies exclusively upon STX4, and STX4 is a rate limiting factor for GLUT4 vesicle fusion to the PM in skeletal muscle.

In the skeletal muscle of diabetic mice, STX4 mRNA levels are significantly reduced (93), suggesting that STX4 deficiency may be important for diabetes development, and that re-expression of STX4 may be a therapeutic strategy to restore insulin sensitivity and reverse T2D. In alignment with this point of view, a recent study showed that enrichment of STX4 (~2 fold) in skeletal muscle remediates peripheral insulin resistance in obese mice, even under conditions of persisting intake of high-fat diet (94). Collectively, these findings highlight the important role and therapeutic potential of STX4 in remediating insulin resistance in skeletal muscle.

Although altering STX4 levels altered skeletal muscle glucose utilization, primary adipocytes of STX4 overexpressing- or knockout (-/+)-mice did not show significant changes in glucose uptake *in vivo* or *ex vivo* (21, 92). Subsequent RNAi studies in 3T3-L1 adipocytes showed that STX4 knockdown reduced insulin-stimulated glucose uptake by 50% (95). While recent STX4 knockout studies, using CRISPR-Cas9 in 3T3-L1 adipocytes, further supported a requirement for STX4 in adipocyte insulin-stimulated glucose uptake *via* GLUT4 translocation (96), an additional role for STX4 in GLUT4 protein stability/biosynthesis was detected. Interestingly, this study detected STX2 and STX3 in 3T3-L1 adipocytes (96); however, the requirement for STX2 or STX3 in the processes of insulin-stimulated GLUT4 translocation was not tested. Given the reported issues with 3T3-L1 adipocytes lacking characteristics of primary adipocytes (97), it will be imperative that inducible adipose-specific STX4 KO mice be evaluated to determine the requirement for STX4 in bona fide adipose tissue in adult mice. Moreover, STX2 or STX3 in skeletal muscle have not been reported. This distinguishing feature of muscle vs. fat cells, in addition to the predominant role of muscle for glucose uptake vs. that of fat, may contribute to these apparent mechanistic differences between these two cell types known to exhibit insulin-stimulated GSV exocytosis.

#### 3.1.2 SNAP23

SNAP23 is a ~23 kDa protein that is associated with the PM *via* palmitoylation of four cysteine residues and is a ubiquitously expressed isoform of SNAP25. Although SNAP23 is expressed in adipose tissue and skeletal muscle, early studies evaluating the role of SNAP23 in GSV translocation were conducted in 3T3-L1 adipocytes. Depletion of SNAP23 using siRNA or interfering with SNAP23 action using botulinum toxin E, peptides encoding the NH2 or COOH termini of SNAP23, and neutralizing antibodies, diminished insulin-stimulated GSV exocytosis in 3T3-L1 adipocytes (12, 95, 98–100). Similarly, overexpression of a SNAP23 mutant (SNAP23-DeltaC8), which specifically precludes binding to VAMP2, suppressed insulin-stimulated GSV exocytosis in 3T3-L1 adipocytes (12). A new mouse model of adipocyte-specific SNAP23 knockout showed peripheral insulin resistance (101), supporting the concept that SNAP23 is physiologically important for glucose uptake into adipocytes *in vivo*. These findings also suggest that SNAP23 deficiency in adipose tissue may contribute to diabetes development. In addition, overexpression of SNAP23 enhanced insulin-stimulated GLUT4 translocation and glucose uptake in 3T3-L1 adipocytes (100). Moreover, SNAP23 protein was three-fold higher in abundance relative to STX4 in 3T3-L1 adipocytes and skeletal muscle (61, 102). Collectively, these findings suggest that SNAP23 can increase GLUT4 vesicle fusion into the PM by enhancing the formation of SNARE complexes with STX4 and VAMP2. Nevertheless, SNAP23 studies conducted in skeletal muscle are needed to determine whether SNAP23 is the only SNAP protein required for GSV exocytosis in skeletal muscle, and performed using an inducible skeletal muscle-specific SNAP23 knockout mouse model.

Clonal β-cells express both SNAP23 and SNAP25, where they reportedly play somewhat interchangeable required roles in glucose-stimulated insulin secretion (103). However, recent *in vivo* studies using a β-cell-specific SNAP23 knockout mouse show that SNAP23 expression limits glucose-stimulated insulin secretion (104). In addition, inhibition of SNAP23 action with a small molecule drug (MF286) increases insulin release from primary islets *ex vivo* (104). Although it is conceivable that SNAP23 may be functionally redundant to SNAP25 for SNARE complex formation, this new recognition that SNAP23 plays a negative role in insulin release in primary pancreatic β-cells suggests that SNAP23 functionality may be cell-type specific. Hence, it implies that developing SNAP23 as therapeutic applications for prediabetes or T2D might be not ideal.

### 3.2 v-SNAREs

In skeletal muscle, multiple v-SNARE isoforms, including VAMP2, VAMP3, VAMP5, and VAMP7, have been identified. VAMP3 knockout mice showed no change in insulin sensitivity or GSV exocytosis in skeletal muscle (105). Early studies in L6 myoblasts pointed toVAMP2 as the primary v-SNARE involved in insulin-stimulated docking and fusion of GSVs with STX4 and SNAP23 (106). During rat muscle contraction *in vivo*, VAMP2, VAMP5 and VAMP7 were also found to translocate to the sarcolemma with GLUT4 (107). *In vivo*, VAMP2 (-/-) knockout mice die immediately after birth whereas VAMP2 (-/+) knockout mice survive but exhibit an abnormal body shape with a round appearance and a shoulder hump (108). Similarly, hippocampal neurons from VAMP2 (-/-) knockout mouse embryos show impaired calcium-triggered fusion of the synaptic vesicle with the presynaptic membrane (108). Therefore, it is hypothesized that VAMP2 is required for GSV fusion *in vivo*, as the vesicle fusion mechanism overlaps between neurons and skeletal muscle, but an inducible skeletal muscle specific VAMP2 knockout mouse model is needed to test this hypothesis and to evaluate therapeutic potential for prediabetes and T2D.

### 3.3 SNARE-Associated Proteins and Post-Translational Modification in GSV Exocytosis

SNARE regulators such as the Munc18 proteins (Munc18-1/Munc18a, Munc18-2/Munc18b, Munc18-3/Munc18c), DOC2 proteins (DOC2A, DOC2B), tomosyn proteins (tomosyn-1, tomosyn-2), and complexin proteins have been shown to play both positive and negative roles in the functionality of SNAREs or SNARE complexes. Recent studies in skeletal muscle have revealed specific expression patterns for select isoforms, which are associated with interesting functions. SNAREs and SNARE regulatory proteins have been reported to be phosphorylated, which modulates SNARE complex formation and exocytosis (109, 110). The role of phosphorylation in SNARE complex formation is summarized in **Table 1**. Examples of SNARE regulators are described in this section below.

**Table 1:** The phosphorylation of SNAREs and SNARE regulators and their effects on vesicle exocytosis.

Protein	Kinase/Phosphatase	Amino Acids (AA)	AA Identification	Mechanism of Action	Reference(s)
STX4	CKII PKA, PKC RAK3D ?	Thr residues ? Ser/Thr residues Tyr115, Tyr251	*In vitro* phosphorylation *In vitro* phosphorylation Mass spectrometry	Decreased binding to SNAP23 Decreased binding to SNAP23 Decreased binding to SNAP23 ?	(111–115)
SNAP23	SNAK PKC PKC PKC IKK	? Ser23, Thr24 Ser161 Ser120, Thr102 Ser95	Mass spectrometry Mass spectrometry Point mutation Point mutation	Modulates SNARE stability and assembly Inhibits interaction with STX4 Minor effect on interaction with STX4 Increases binding to STX4 and VAMP2 Increases binding to STX4 and VAMP2	(116–120)
VAMP2	CAMKII CKII PKCζ	Thr35, Ser61 Ser75 Ser residues	Prediction algorithm Prediction algorithm *In vitro* phosphorylation	? ? Promotes insulin-stimulated GLUT4 translocation	(121–124)
Munc18c	Insulin receptor/PTP1B ?	Tyr218, Tyr219, Tyr521 Thr569	Prediction algorithm Prediction algorithm	Dissociates from STX4 No effect on Munc18c association with STX4	(115, 125–129)
DOC2B	PKC iota Src ?	Ser34 Tyr301 Tyr301	Prediction algorithm Prediction algorithm Prediction algorithm	Promotes DOC2B translocation to the plasma membrane Binds ERM proteins and promotes insulin release (β-cells) Binds KLC1 and promotes insulin-stimulated GLUT4 translocation in skeletal muscle cells	(130–132)
Tomosyn	AKT1, AKT2	Ser783	Prediction algorithm	Dissociates from STX4	(133)
Synip	AKT2	Ser99	Point mutation	Dissociates from STX4	(134–137)

The specific sites of phosphorylation on SNARE and SNARE regulators were identified using in vitro phosphorylation assay, point mutation assay, computational algorithm prediction, or mass spectrometry. The kinases or phosphatase for the target proteins were identified by in vitro binding assays. The question mark (?) denotes that the identity of the kinase or phosphatase is unknown.

#### 3.3.1 Munc18c

**Figure 2:**
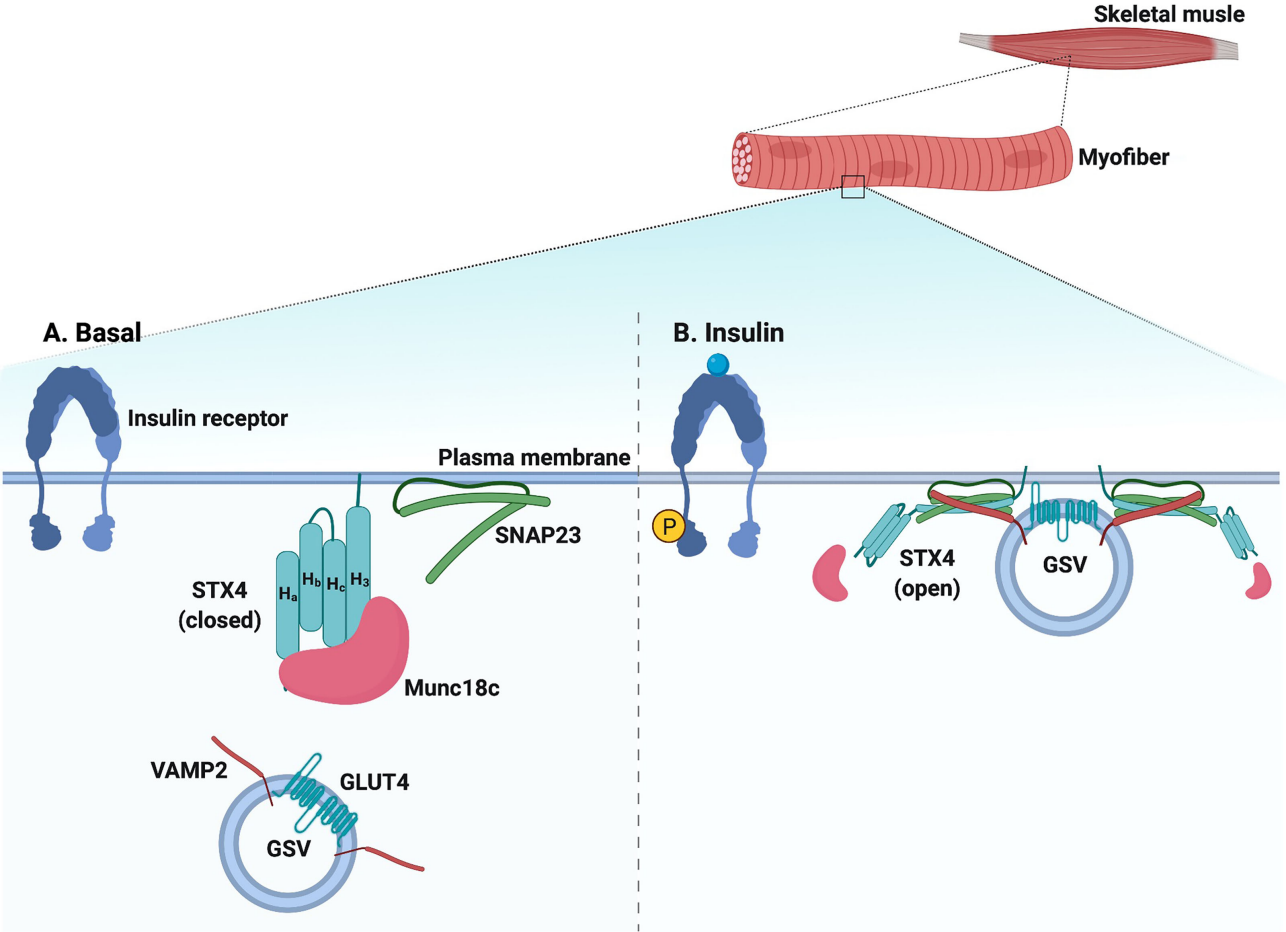
Schematic diagram of SNARE-mediated GSV translocation in skeletal muscle. **(A)** Under basal conditions, SNARE proteins (VAMP2 on the GLUT4 storage vesicle [GSV] and t-SNAREs STX4 and SNAP23 on the plasma membrane [PM]) are present in monomeric conformations with STX4 in a closed conformation. Munc18c is bound to STX4, precluding SNARE complex assembly. **(B)** In response to insulin, Munc18c may be bound in a low affinity interaction to the N-terminal peptide region of STX4; data also supports Munc18c dissociating from STX4, leading to an open STX4 structure that mediates SNARE complex formation, GSV docking, and fusion with the PM. Created with BioRender.com and licensed for publication.

The Munc18 family of proteins, also called Sec1/Munc18 (SM) proteins, are the most extensively studied regulators of SNARE complex formation in GSV exocytosis. The Munc18 family members are 66-68 kDa soluble proteins, and while they lack an apparent transmembrane domain, they are localized to the PM through direct interaction with their cognate STX partners (138, 139). Mammalian cells express Munc18a, Munc18b, and Munc18c; however, only Munc18b and Munc18c are expressed in the skeletal muscle and adipocytes (138). Of these two, it is only Munc18c that binds to STX4 with high affinity (140) (**Figure 2**).

Munc18c is agreed to be an essential regulator for STX4-based SNARE complex assembly and fusion to the PM, but the detailed mechanisms involved have been a challenge to gain consensus amongst labs using different knockout mouse models, RNAi, detection antibodies, and cellular testing systems. For example, while consensus exists that overexpression of Munc18c in 3T3-L1 adipocytes inhibits insulin-stimulated GSV translocation and glucose uptake (56, 59), the outcome in RNAi-depleted cells or knockout mice is less clear. For instance, one strain of Munc18c knockout(-/+) mice ([-/-] mice die by E7.5) on a C57Bl6 background showed impaired insulin-stimulated GSV translocation in skeletal muscle, indicating that Munc18c is required for insulin-stimulated GSV translocation and glucose uptake (17), with no impact in adipose tissue; this phenotype was reproduced by others (141). By contrast, a second Munc18c knockout (-/-) mouse strain showed an unexpected gain of function response in the adipose tissue (142). However, in this case, they studied the few Munc18c (-/-) knockout mice that survived, and adipose tissue but not skeletal muscle function was assessed; no other studies of this mouse model are reported. Inducible adipose tissue-specific and skeletal muscle-specific Munc18c knockout mouse models are needed to investigate target tissue-specific insulin-stimulated GSV translocation and glucose uptake *in vivo* and to evaluate a candidate therapeutic target for prediabetes and T2D.

Intriguingly, the negative effect of overexpressing Munc18c is associated with relative localized levels of STX4 in muscle cells. Overexpression of Munc18c *via* adenovirus injection in skeletal muscle inhibited GSV translocation to the t-tubule membrane, yet was without adverse effects on GLUT4 translocation to the sarcolemma membrane. Increased STX4 expression in the sarcolemmal membrane, compared to less in the t-tubule membranes, correlated with resistance to the negative effects of Munc18c in the sarcolemma vs. t-tubules (143). Consistent with this, overexpression of STX4 reversed the inhibitory effect of Munc18c overexpression on insulin-stimulated GSV exocytosis in 3T3-L1 adipocytes (144). This finding suggests that the local balance of STX4:Munc18c is important for optimal GSV exocytosis. Although the molecular details of the Munc18c-regulated SNARE complex assembly remain incomplete, there is consensus that insulin can alter the mode of interaction between Munc18c and STX4, enhancing GLUT4 translocation (6, 145). For example, phosphorylation of Munc18c results in its dissociation from STX4, perhaps switching its affinity for an alternate binding partner, DOC2B, leading to STX4 activation and SNARE assembly (20, 125, 126, 140). The roles of Munc18c phosphorylation and DOC2B will be described in the next section.

#### 3.3.2 DOC2B

Growing evidence suggests that DOC2B is essential for SNARE complex assembly and enhances GSV exocytosis in skeletal muscle and adipocytes (20, 58, 146, 147). DOC2B is a ubiquitously expressed 46-50 kDa protein harboring an N-terminal Munc13-interacting domain and C-terminal tandem calcium- and phospholipid-binding C2 domains (C2A and C2B) (148). The C2B domain plays a central role in the DOC2B interaction with Munc18c (149); DOC2B appears to serve as a scaffold for transient binding of Munc18c to facilitate SNARE complex assembly and GLUT4 translocation. Consistent with this, skeletal muscle from DOC2B knockout (-/-) mice shows increased abundance of Munc18c-STX4 complexes (20). Indeed, multiple labs consistently report that DOC2B knockout mice exhibit glucose intolerance (20, 150). This is linked to impaired insulin-stimulated GLUT4 accumulation in the sarcolemmal membrane, as determined using differential ultracentrifugation to subfractionate freshly isolated muscle, and it correlates with reduced SNARE complex formation in muscle (20). However, the result is controversial because impaired GLUT4 translocation and glucose transport was reproduced in DOC2B knockout mouse adipocytes but not muscle in a study using tissue explants *ex vivo* (150). It remains to be determined whether methodological differences in the muscle studies may have contributed to the discrepant outcomes.

In contrast, mice globally overexpressing DOC2B show enhanced skeletal muscle insulin-stimulated GLUT4 translocation and SNARE complex assembly, along with decreased Munc18c:STX4 interaction in skeletal muscle (58). A recent report shows that skeletal muscle-specific overexpression of DOC2B, induced in adult mice, phenocopies the global DOC2B overexpressing mice (132), indicating that skeletal muscle plays critical role in driving whole-body insulin sensitivity. Furthermore, skeletal muscle-specific DOC2B overexpression, induced in adult mice, protects against high-fat diet-induced insulin resistance, even when obesity persists (132). In line with the concept that DOC2B acts as a positive regulator of insulin sensitivity, it has been reported that DOC2B can induce membrane curvature during the fusion of synaptic vesicles by penetrating its C2A and C2B domains into the PM (151). Typically, the PM is a flat membrane in the basal state. The simultaneous insertion of proteins bearing two C2 domains like DOC2B into the PM together with calcium induces membrane curvature that provides the energy to assemble the SNARE complex, resulting in sufficient fusion (152). Interestingly, DOC2B levels are reduced in T2D human skeletal muscle (132), suggesting that DOC2B loss could be important for diabetogenesis and implicating DOC2B as a candidate therapeutic target for prediabetes and T2D.

#### 3.3.3 Complexin-2

The complexin proteins were first identified as regulatory proteins involved in synaptic vesicle exocytosis in neurons (153, 154). Recently, the expression of complexin-2 was also identified in skeletal muscle cells (62). Complexins contain an N-terminal accessory helix and a central helix, which interacts with the ternary SNARE complex in a calcium-dependent manner (155, 156). In skeletal muscle cells, complexin-2 translocates to the PM *via* the insulin-induced activation of the Rho-family GTPase Rac1 in the noncanonical insulin signaling pathway (62). Depletion of complexin-2 using siRNA inhibits insulin-stimulated GLUT4 accumulation at the L6 GLUT4myc myoblast surface (62), but overexpression of complexin-2 also impairs insulin-stimulated GLUT4 translocation (62). These findings suggest that complexin-2, similar to Munc18c, is needed in a precise quantity in muscle cells for GSV exocytosis. Factors such as Munc18c and complexin-2, which have narrow windows of cellular tolerance, may be challenging as therapeutic targets for prediabetes and T2D.

#### 3.3.4 Tomosyn

Tomosyn is a 130 kDa protein initially purified from brain and identified as a t-SNARE binding protein that negatively regulates SNARE-dependent neurotransmitter release (157–160). Structurally, tomosyn proteins possess 14 WD40 repeats arranged in two seven-bladed propeller domains and a C-terminal domain that contains multiple predicted phosphorylation sites and harbors a v-SNARE-like motif (161–163). Both the N- and C-terminal domains of tomosyn play distinct roles in SNARE association. Mechanistically, the N-terminal domain of tomosyn is associated with binding to the STX monomer (either STX4 or STX1), whereas the C-terminal domain of tomosyn competes with VAMP2 *via* tomosyn’s v-SNARE-like motif, preventing the initiation of ternary SNARE complex assembly (57, 133). Overexpression of tomosyn (b-tomosyn) in 3T3-L1 adipocytes inhibits insulin-stimulated GLUT4 translocation (60). Consistent with this observation, knockout of the tomosyn isoforms expressed in adipocytes (tomosyn-1 and tomosyn-2) increases GSV exocytosis under both basal and insulin-stimulated conditions (164). Further, it was shown that tomosyn shares the same mechanism of action in adipocytes as in neurons, in which tomosyn binds to partially assembled t-SNARE complexes and arrests membrane fusion (164). While tomosyn inhibitors would seemingly be attractive therapeutics to increase insulin-stimulated glucose uptake by muscle, the potential for elevation of basal glucose uptake, causing hypoglycemia, would require careful consideration as a therapeutic target for prediabetes and T2D.

#### 3.3.5 Synip

Synip (62 kDa) is a STX4-interacting protein first identified from a 3T3-L1 adipocyte library yeast two-hybrid screen (55). Mechanistically, Synip competes with the v-SNARE VAMP2 for binding to STX4, yet it does not affect the ability of SNAP23 to bind STX4. In 3T3-L1 adipocytes, Synip binds to STX4 under basal conditions, and in response to insulin, the N-terminus of Synip dissociates, permitting STX4 engagement in GSV fusion (55). Intriguingly, the C-terminal half of Synip remains anchored to the PM by binding to PIP_3_ *via* its WW domain (165). Although skeletal muscle expresses abundant Synip mRNA, it remains to be investigated whether Synip plays a similar role in GSV exocytosis and glucose uptake in skeletal muscle.

#### 3.3.6 VAP-33

Vesicle-associated protein 33 (VAP-33) is implicated as an inhibitory regulator of GSV exocytosis in L6 skeletal muscle cells and 3T3-L1 adipocytes (166). VAP-33 has a single-transmembrane domain and binds to VAMP2 (167). Overexpression of VAP-33 diminished insulin-stimulated GLUT4 translocation, and this effect was restored by co-overexpression of VAMP2 in L6 skeletal muscle cells (166). It is possible that excess VAP-33 prevents either the entry of VAMP2 into the t-SNARE complex or subsequent vesicle fusion (166). Further studies are necessary to elucidate how VAP-33 affects SNARE complex formation and the fusion machinery.

#### 3.3.7 Munc18c Phosphorylation and STX4 Interaction

**Figure 3:**
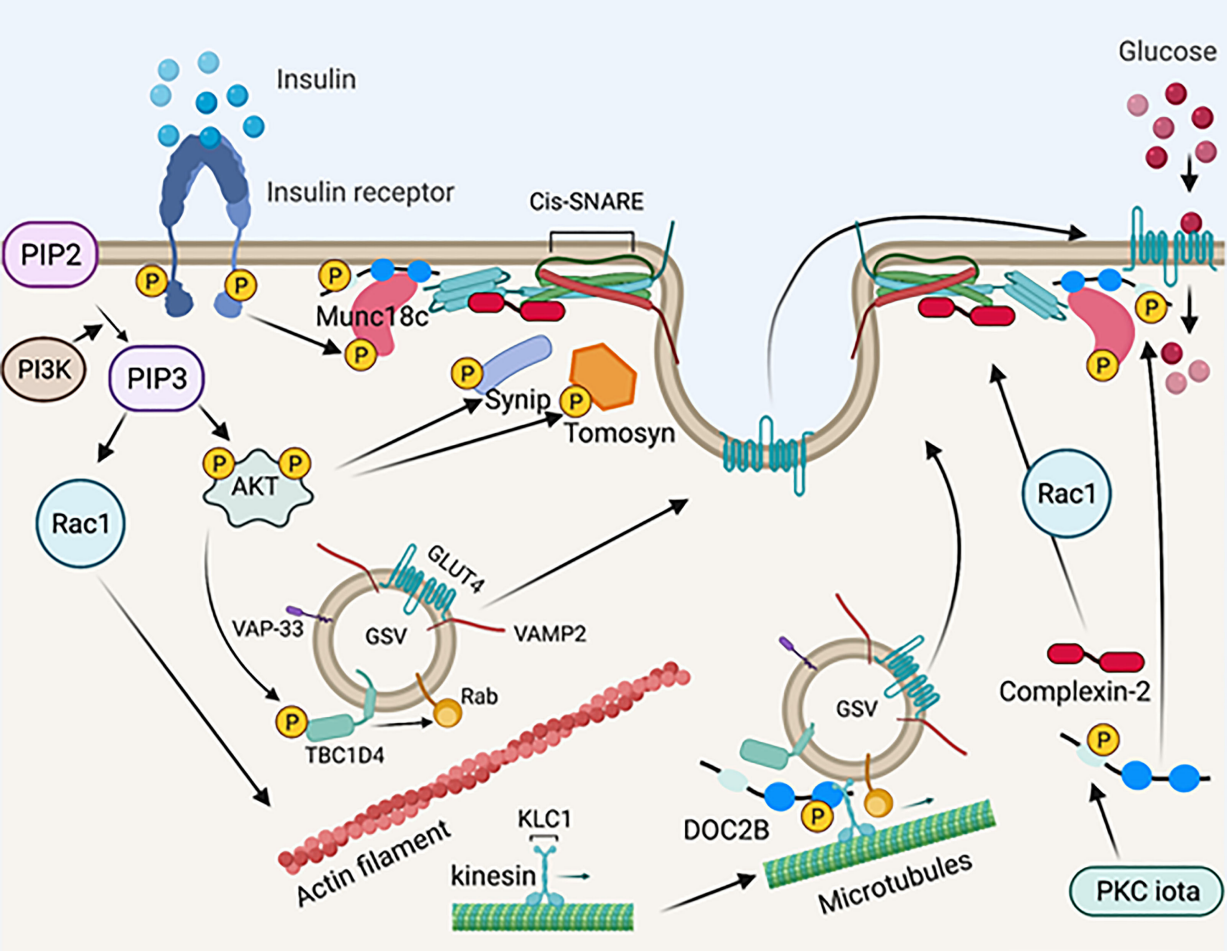
Schematic diagram of SNARE-mediated membrane fusion *via* post-translational modification. Upon insulin stimulation, the insulin receptor phosphorylates Munc18c. Phosphorylated Mun18c transiently switches its binding from STX4 to DOC2B. Simultaneously, the insulin receptor is auto-phosphorylated and recruits PI3K, resulting in phosphorylation and activation of AKT. Activated AKT phosphorylates Synip and tomosyn, which results in its dissociation from STX4. These events induce the formation of a four-helix bundle cis-SNARE complex, which facilitates GSV fusion with the PM and inserts GLUT4 on the PM for glucose uptake. Activated AKT also phosphorylates TBC1D4, which directly acts on Rab GTPases on the GSV and leads to GLUT4 mobilization and/or fusion. PI3K recruits Rac1, which results in actin remodeling and permits GLUT4 translation to the PM. Meanwhile, DOC2B is phosphorylated by PKC iota and is redistributed from the cytosol. DOC2B phosphorylation also facilitates interaction of the GSV with microtubule protein KLC1, leading to GLUT4 mobilization toward the PM. Insulin stimulation also induces Rac1 dependent complexin-2 translocation to the PM, stabilizing the SNARE complex and regulating GSV fusion. Created with BioRender.com and licensed for publication.

In response to insulin, Munc18c is directly phosphorylated on Tyr219 and Tyr521 by the insulin receptor tyrosine kinase (115, 125–128). Interestingly, the phosphorylation of Munc18c occurs rapidly and is independent of PI3K activation (125, 128), suggesting that Munc18c functions in parallel with IRS-1 activation, coordinating SNARE activation at the PM with the timed arrival of GLUT4 vesicles mobilized in response to insulin signaling. Insulin-stimulated Munc18c phosphorylation triggers its dissociation from STX4, and phospho-defective mutants of Munc18c (Tyr219Phe and Tyr521Phe) fail to dissociate from STX4 upon insulin stimulation (125, 128, 129). In contrast, phospho-mimetic mutants of Munc18c (Tyr219Glu and Tyr512Glu) dissociate from STX4 in the absence of insulin (125, 128), demonstrating the functional significance of Munc18c phosphorylation in insulin-stimulated GSV exocytosis. The tyrosine phosphorylation of Munc18c induces a switch in binding specificity from STX4 to DOC2B (125, 140), as has been observed in 3T3-L1 adipocytes and in primary muscle (58) (**Figure 3**). Furthermore, the Munc18c Tyr219 and Tyr512 residues are located within non-structured surface-exposed domains (47) and hence have the potential to interact with other regulatory proteins. In line with this, Munc18c is reportedly a target for protein-tyrosine phosphatase 1B (PTP1B) in adipocytes, which can regulate Munc18c phosphorylation status (129). Hence, targeting the phosphorylation status of Munc18c may provide a therapeutic opportunity to increase insulin-stimulated glucose disposal by muscle or adipose tissue. It will be of key importance to investigate the phosphorylation status of Munc18c under physiologically relevant conditions such as nutritional or metabolic stress, prediabetes, and T2D.

#### 3.3.8 DOC2B Phosphorylation and Microtubule Interaction

In addition to interacting with insulin-induced tyrosine-phosphorylated Munc18c in the “switch mechanism” described above, DOC2B was recently shown to undergo insulin-induced tyrosine phosphorylation at Tyr301, located in the DOC2B C2B domain (132). In islet β-cells, phosphorylation of DOC2B at Tyr301 occurs rapidly, serving as the substrate for the glucose-activated Src tyrosine kinase; phosphorylated DOC2B then associates with ERM proteins to trigger downstream insulin granule mobilization to support insulin release (131). In L6 skeletal muscle cells, phosphorylation at Tyr301 on DOC2B triggers DOC2B association with the microtubule protein kinesin light chain 1 (KLC1) (132). Microtubule proteins are essential for GSV mobilization to the PM in response to insulin stimulation (168–170). Importantly, while overexpression of wild-type DOC2B in L6 skeletal muscle cells prevented insulin resistance, a phosphodeficient mutant of DOC2B, Tyr301Phe, failed in this regard (132). In 3T3-L1 adipocytes, insulin stimulates DOC2B phosphorylation at Ser34 *via* the atypical PKC iota, which promotes DOC2B translocation to the PM (130). Whether PKC iota is a functional kinase in muscle, and whether both Tyr301 and Ser34 undergo phosphorylation in primary muscle, remains unknown in the absence of DOC2B phosphorylation site-specific antibodies are available. In addition, it remains to be determined whether the pool of DOC2B protein that complexes with tyrosine-phosphorylated Munc18c at the PM, also participates in the microtubule mobilization of GSVs, or translocates to the PM in response to insulin. Given the rapid tyrosine phosphorylation of DOC2B in response to insulin (5 minutes), it is conceivable that these phosphorylation events and interactions occur in different cellular pools of DOC2B (**Figure 3**).

#### 3.3.9 Tomosyn Phosphorylation and STX4 Interaction

Tomosyn (b-tomosyn-1) binds to STX4, and phosphorylation of tomosyn at Ser783, *via* Akt, inhibits tomosyn:STX4 binding (133). Consistent with this, a phospho-mimetic mutant tomosyn (Ser783Asp) reduced tomosyn binding to STX4 (133). Mutation of Ser783 (Ser783Ala) results in constitutive tomosyn:STX4 binding, which correlates with attenuated insulin-stimulated glucose uptake in C2C12 skeletal muscle cells (133). Hence, these findings suggest that tomosyn phosphorylation could be targeted to improve insulin-stimulated glucose uptake into skeletal muscle (**Figure 3**).

#### 3.3.10 SNARE Protein Phosphorylation: VAMP2 and STX4

Three phosphorylation sites (Thr35, Ser61 and Ser75) on VAMP2 have been reported in synaptic vesicles, each located within a SNARE motif. Thr35 and Ser61 are phosphorylated by CaMKII, whereas CKII phosphorylates Ser75 (121–123). Nevertheless, the physiological significance of VAMP2 phosphorylation and whether phosphorylation of VAMP2 modulates its ability to associate with other SNARE proteins has not been demonstrated. Serine phosphorylation of VAMP2 in primary rat skeletal muscle occurs in response to insulin, *via* PKCζ (124). PKCζ co-localizes with GSVs, and its overexpression drives GLUT4 translocation to the PM and glucose transport even in the absence of insulin, acting as an insulin-mimetic; however, overexpression of a dominant-negative mutant of PKCζ abrogates these effects (124). Hence, these findings suggest that VAMP2 phosphorylation is important for insulin-stimulated GLUT4 translocation.

Although early proteomic studies revealed STX4 phosphorylation at Tyr115 and Tyr251 in response to insulin in 3T3-L1 adipocytes (115), this has yet to be validated to date, either in adipocytes or skeletal muscle. In cancer cells and islet β-cells, STX4 has a putative phosphorylation site at Ser78, which when mutated to Ser78Ala, results in STX4 protein stabilization (171, 172) Given the pivotal importance of STX4 in skeletal muscle glucose uptake, capitalizing on this stabilizing mutation could harbor therapeutic potential.

## 4 Atypical Roles of SNARE Proteins

### 4.1 Contribution to Mitochondrial Dynamics

STX4 was previously thought to localize only to the PM, where it assembles with VAMP2 to dock and fuse GSVs to the PM. Interestingly, STX4 was recently reported to localize to various intracellular organelles and compartments, including the mitochondrial membranes of multiple cell types (173). Mitochondria are not only the powerhouses of cells, but they also play a vital role in skeletal muscle metabolism. Thus, mitochondrial dysfunction is directly linked to a variety of disorders, including insulin resistance (174, 175), muscular dystrophies (176), and sarcopenia (177). Mitochondria continuously cycle between fission and fusion in a process referred to as mitochondrial dynamics. Mitochondrial fission entails separating dysfunctional mitochondria, and replacing them with daughter mitochondria, thereby improving the mitochondrial network and mitochondrial function (178). However, overly high rates of mitochondrial fission lead to pathophysiological conditions such as sarcopenia (177). In contrast, mitochondrial fusion regulates mitochondrial DNA replication, mitochondrial nucleotide distribution, and oxidative phosphorylation capacity (179–183). Skeletal muscle-specific STX4 enrichment tips the balance in favor of increased mitochondrial fusion in mice. High-fat diets are known to increase mitochondrial fission and mitophagy (184), a hallmark of high-fat diet-induced diabetes. STX4 enrichment reversed both skeletal muscle insulin resistance and mitochondrial fragmentation and dysfunction in high-fat diet-fed mice (94). The mechanism is currently believed to involve suppression of mitochondrial fission.

Mitochondrial fission is largely modulated by dynamin-related protein (Drp1) (185), and STX4 enrichment in skeletal muscle reduced Drp1 protein abundance, which would be associated with the elongated mitochondrial structure found in the skeletal muscle of skeletal muscle-specific STX4 overexpressing mice.

**Figure 4:**
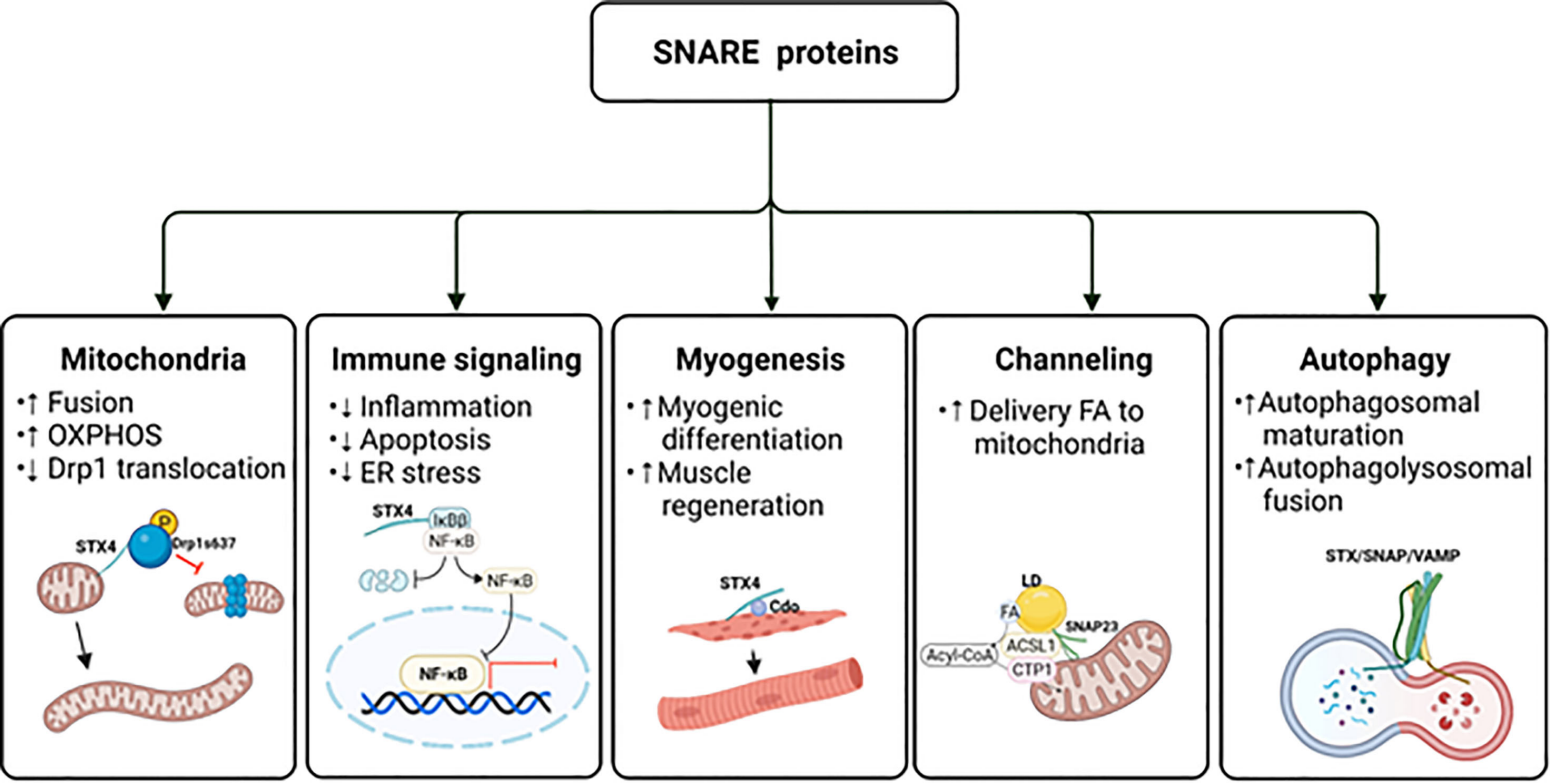
The potential impact of SNAREs beyond GLUT4 vesicle exocytosis. The schematic diagram provides an overview of the atypical actions of SNARE proteins. ER, endoplasmic reticulum; FA, fatty acid; LD, lipid droplet; OXPHOS, oxidative phosphorylation. Created with BioRender.com and licensed for publication.

In addition, Drp1 undergoes phosphorylation, which regulates mitochondrial fission. Specifically, phosphorylation of Drp1 at Ser616 promotes mitochondrial fission by facilitating Drp1 translocation from the cytosol to the mitochondria (186), whereas phosphorylation of Drp1 at Ser637 inhibits its translocation, thereby promoting mitochondrial fusion (187). In primary skeletal muscle and L6 skeletal muscle cells, STX4 binds to Drp1 and promotes its phosphorylation at Ser637, but not Ser616, through a mechanism involving AMPK (94). In addition, knockdown of STX4 reduces Drp1 phosphorylation at Ser637 and AMPK activation (**Figure 4**). Further studies are necessary to reveal the structural motif of STX4 that mediates the association with Drp1 and to determine how STX4 migrates to the mitochondria in skeletal muscle. Finding avenues to harness the positive effects on STX4 on mitochondrial dynamics in skeletal muscle carries tantalizing potential for therapeutic benefit.

### 4.2 Contribution to Immune Signaling

Elevated levels of the proinflammatory cytokine TNF-α promote skeletal muscle insulin resistance (188). Indeed, obese ob/ob mice deficient in TNF-α receptors (TNFRs) exhibit improved insulin sensitivity (189). Molecularly, extracellular TNF-α binds to and activates the PM-localized TNFR, thereby activating the IκB kinase (IKK) complex composed of catalytic kinase subunits (IKKα and IKKβ) and a non-catalytic regulatory subunit (NEMO/IKKγ), which phosphorylates IκBs. The phosphorylation of IκBα and IκBβ leads to proteasomal degradation, releasing the IkB’s hold on cytosolic NF-κB, resulting in NF-κB translocation to the nucleus. Nuclear NF-κB transactivates genes encoding pro-inflammatory mediators, leading to the pathogenesis of insulin resistance and muscle atrophy. The IKKβ aspect of the NF-κB pathway in particular is linked to the development of insulin resistance (190); inhibition of canonical IKKβ/NF-κB signaling actually improves skeletal muscle insulin sensitivity (191, 192) and prevents muscle degeneration and myofiber death (193–197).

Recently, STX4 in pancreatic islet β-cells was found to selectively reduce TNFα-driven IKKβ signaling (198). Specifically, STX4 binds to the IKKβ target, IκBβ, preventing its phosphorylation and subsequent proteasomal degradation, which reduces the translocation of NF-κB into the nucleus. This prevents proinflammatory cytokine-induced expression of chemokine ligands CXCL9 and CXCL10 (199), creating more resilient islet β-cells (**Figure 4**). Remarkably, the IKKα-IκBα-specific signaling arm was entirely unaffected by STX4. Resilience was further improved by overexpression of a phosphorylation-resistant Ser78Ala mutant STX4. Moreover, single-cell RNA sequencing analysis of mouse islets isolated from inducible β-cell-specific STX4-overexpressing non-obese diabetic mice (NOD-iβSTX4) further validated the role of STX4 in regulating immune signaling. Specifically, NOD-iβSTX4 mouse islets showed reduced interferon-γ signaling and TNFα signaling *via* NF-κB (200). Given that upregulated NF-κB signaling is implicated in the pathogenesis of insulin resistance, avenues to target selectively the IκBβ cascade, leaving the IκBα arm intact, perhaps using a stabilized STX4, could provide a nuanced therapeutic means to limit NF-κB-mediated insulin resistance, muscle degeneration and myofiber death.

In addition to STX4, DOC2B confers protection against inflammatory damage in β-cells *in vivo*. DOC2B knockout (-/+) mice are highly vulnerable to streptozotocin, resulting in increased β-cell apoptosis and loss of β-cell mass (201), while inducible β-cell-specific DOC2B overexpressing mice resist streptozotocin-induced β-cell apoptosis and loss of β-cell mass (201). DOC2B enrichment in β-cells also ameliorates cytokine-induced apoptosis and endoplasmic reticulum stress, and a truncated DOC2B protein, which contains only the two C2 domains, is sufficient to confer this resilience (201). Additionally, phosphorylation at Tyr301 within the C2B domain of DOC2B confers protection against insulin-resistance-inducing stress to skeletal muscle cells (132), as discussed in Section 3.3.8. These findings suggest the presence of atypical mechanistic crosstalk between typical exocytosis proteins and inflammatory pathways.

### 4.3 Contribution to Myogenesis

Myogenesis is a well-coordinated multi-step process that forms multi-nucleated myofibers by cell fusion; it is also crucial for muscle regeneration and maintenance (202). Recent research has reported that STX4 plays a role in myogenesis *via* its interaction with cell adhesion molecule-related/down-regulated by oncogenes (CDO) (203), which is a multifunctional cell surface receptor located at cell-cell contact sites (204) that regulates myogenic differentiation. Embryos from CDO knockout (-/-) mice are defective in muscle development (205), and primary myoblasts isolated from CDO knockout mice show defective differentiation and deficient activity of p38MAPKα/β (206). In contrast, overexpression of CDO accelerates myogenic differentiation in C2C12 myoblasts (207). Overexpression or depletion of STX4 in C2C12 myoblasts promotes or inhibits myogenic differentiation, respectively, by regulating CDO and p38MAPK (203). Interestingly, STX4 protein abundance also increases progressively during C2C12 myoblast differentiation (203). STX4 confers pro-myogenic function by physically interacting with CDO *via* the SNARE motif of STX4, which regulates the cell surface localization of CDO during differentiation (203). Furthermore, CDO is required for STX4-mediated GLUT4 vesicle fusion at the PM during myoblast differentiation (203). Collectively, these findings suggest that STX4 stimulates translocation of CDO, which in turn activates p38MAPK, which induces myogenic differentiation in skeletal muscle cells.

The nutritional environment is important for myogenesis. For example, an abnormal nutritional environment inhibits myoblast differentiation and decreases the number of myofibers (208–212). Given that deficits in STX4 protein abundance are associated with diabetes, and that the STX4 gene has been implicated as a genetic factor related to obesity-induced metabolic disease development in humans (213), it is possible that abnormal nutritional environment could decrease myogenesis and promote sarcopenia. Conceptually, it follows that STX4 enrichment could block the negative effects of abnormal nutrition. Indeed, overexpression of STX4 in insulin-resistant mice promoted normal striated muscle structure, correlated with resumption of voluntary exercise, despite intake of a nutritionally deleterious high-fat diet (94). This suggests that enhancing STX4 function might be a therapeutic strategy to support myogenesis even in an insulin-resistant environment (**Figure 4**).

VAMP2 is expressed in muscle satellite cells, which are quiescent myogenic cells that do not express GLUT4, and in myogenic cells during embryonic development (214, 215). Increased VAMP2 expression was observed during muscle regeneration after muscle injury by snake venom, cardiotoxin, suggesting that VAMP2 may regulate muscle regeneration (214). To date only one study, wherein miR-127-3p was shown to induce myogenesis by targeting the VAMP2 gene, provides mechanistic insight into how VAMP2 regulates myogenesis (216). Additional mechanistic details linking VAMP2 to myogenesis remain to be elucidated.

### 4.4 Contribution to Lipid Droplet Fusion and Channeling of Fatty Acids Into Mitochondria

Although SNAP23 is traditionally thought to participate in exocytic vesicle docking at the PM, recent studies have implicated SNAP23 in divergent roles in lipid droplet fusion, and in the channeling of lipid droplet-derived fatty acids into mitochondria for β-oxidation. Lipid droplets are dynamic cellular lipid-storage organelles that function as lipid reservoirs for energy production (217). In skeletal muscle, lipid droplets reside in the subsarcolemmal region or in-between the myofibrils, where they provide substrates for energy production (218). SNARE proteins were found localized to lipid droplets, and lipid droplet fusion was positively associated with SNAP23, STX5 (anterograde ER–Golgi transport), and VAMP4 protein abundances (219). Deletion of SNAP23, STX5, or VAMP4 reduced the fusion rate and size of lipid droplets, indicating a requirement for these SNARE isoforms for lipid droplet fusion. In HL-1 cardiac muscle cells, an increase in the lipid droplet pool sequestered SNAP23 away from the PM to the intracellular lipid droplets, where SNAP23 contributed to lipid droplet growth through lipid droplet fusion (219). A recent study applying immunofluorescence microscopy showed that SNAP23 colocalized with PM as well as with mitochondria and not as much with lipid droplets in lean human skeletal muscle (220). This finding also suggests that SNAP23 could be redistributed from the PM to lipid droplets during lipid oversupply in obese individuals, which causes reducing GSV exocytosis, contributing to dysfunctional insulin-stimulated glucose uptake in skeletal muscle (221).

In addition to its contribution to lipid droplet fusion, SNAP23 also mediates lipid droplet-mitochondria interactions, channeling fatty acids released by lipid droplets into the mitochondria. Deletion of SNAP23 reduces complex formation containing mitochondria and lipid droplets, and decreased mitochondrial fatty acid β-oxidation in NIH 3T3 fibroblasts (222). Furthermore, lipid droplet-mitochondria interactions increase in response to starvation in hepatocytes (223). A recent study reported interaction between SNAP23 and acyl-CoA synthetase long chain family member 1 (ACSL1) on the mitochondria during glucose deprivation in primary hepatocytes, linked to enhanced fatty acid oxidation (224) (**Figure 4**). In skeletal muscle, insulin sensitivity strongly influences the capacity to increase fatty acid oxidation. The accumulation of harmful intermediates due to impaired fatty acid oxidation has been associated with insulin resistance (225). Although SNAP23 has been found in mitochondria of human skeletal muscle, there are no reports to date regarding SNAP23-associated fatty acid oxidation. Given that SNAP23 functions in the channeling of fatty acids into mitochondria in skeletal muscle, such a finding could reveal new options for treating metabolic disease.

### 4.5 Contribution to Autophagy

Autophagy is an essential intracellular degradation process that results in the clearance of cell debris or damaged organelles. Macroautophagy is the most prevalent autophagic process that leads to sequestering compartments such as damaged organelles, cytosolic proteins, and invasive microbes within cytosolic double-membrane vesicles (226). Autophagic dysfunction has been reported to be associated with diabetes (227–229) and autophagy-related genes and proteins are decreased in skeletal muscle biopsies from insulin-resistant patients with T2D (230). Consistent with this, upregulation of autophagy enhances insulin sensitivity (231–233). Recent studies have implicated the SNARE proteins SNAP23 (yeast Sec9) and STX1 (yeast Sso2p) as essential for the recruitment of autophagy components and autophagosome biogenesis in yeast (234).

In mammalian cells, autophagic activity requires the v-SNARE VAMP7 and t-SNARE STX7, STX8, and the protein Vti1b (Vesicle transport through interaction with t-SNAREs homolog 1B) (235). Inhibition of SNARE protein-dependent fusion reduces the size of autophagic precursors, which inhibits their maturation into autophagosomes. In addition, the SNARE proteins STX17, SNAP29, and VAMP8 mediate the final step of autophagy, autophagolysosomal fusion (235). VAMP2 homolog YKT6, an R-SNARE, has also been identified as a secondary SNARE protein capable of mediating STX17-dependent autophagolysosomal formation (236). Moreover, a recent study demonstrated that SNAP23 can control macroautophagy through an autophagy-related protein 9 (ATG9)-dependent pathway, which regulates the protein levels and activation of the proapoptotic regulator BCL-2-associated X protein (BAX) (101).

In addition to conventional degradative role of autophagy, autophagy plays a role in unconventional cytosolic secretion of autophagy cargo, such as IL-1β. This process takes place through secretory autophagy cargo receptor tripartite motif-containing protein 16 (TRIM16) and R-SNARE Sec22b in combination with STX3 and STX4, and SNAP23 and SNAP29 (237). This finding suggests that secretory autophagy is mediated by cargo receptors and PM SNARE proteins (237). Altogether, these reports emphasize atypical roles for SNARE proteins that extend beyond traditional GSV exocytosis (**Figure 4**).

## 5 Concluding Remarks and Perspectives

Considerable progress has been achieved in understanding GSV exocytosis over the past three decades. Particularly, the discovery of the mechanisms by which GLUT4 is incorporated into the PM *via* the STX4-based SNARE complex in response to insulin stimulated has received tremendous attention because it plays a pivotal role in regulating glucose uptake and clearance from the circulation to restore normoglycemia. As a result of this important therapeutic potential, research on SNARE proteins, including both typical and atypical functions, has moved rapidly in the last few years. This review has attempted to shed light on recent key discoveries and their potential therapeutic applications.

While key features of SNAREs and their regulators have been well established, numerous mechanistic and integrative aspects of their function remain to be elucidated. For example, the phosphorylation of DOC2B at Tyr301 is crucial for interacting with myotubules during GLUT4 vesicle mobilization to the PM (132), but the specific kinase has not been identified in skeletal muscle cells. Of note however, the kinase targeting Tyr301 site of DOC2B in islet β-cells has just been identified as YES kinase, a rapidly acting glucose-responsive Src family tyrosine kinase (131). It will be important to determine if this is a conserved mechanism between β-cells and skeletal muscle cells going forward. In addition, it remains elusive whether phosphorylation of DOC2B affects its interaction with Munc18c, which regulates the conformational change of STX4 to facilitate GSV exocytosis. Moreover, the extent of individual and combined phosphorylation of SNAREs and their regulators in prediabetes and T2D remains largely unknown and phospho-specific antibodies for SNARE and SNARE accessory proteins are rare (pTyr219-Munc18c is the only known phospho-specific antibody (125). Furthermore, it is not known whether other post-translational modifications of SNAREs and their regulatory proteins contribute to the function of the SNARE complex in skeletal muscle. Although the entire crystal structures of SNAREs and their regulatory proteins in various conformational changes are not completely resolved to date, the basic motifs and enzymes that phosphorylate or dephosphorylate SNAREs and their regulatory proteins are actively being elucidated. Hence, using this information, understanding the three-dimensional structure and conformation changes by post-translational modification would be valuable to enhance our knowledge about the effect of post-translational modification on SNARE function.

Overall, there is increasing evidence of the importance of SNAREs and their regulators for insulin sensitivity. Because treatments for prediabetes and T2D aim to enhance peripheral insulin sensitivity, it will be paramount to elucidate the various physiological roles and regulation of SNAREs and their regulators. These insights will provide ways to combat impaired insulin sensitivity and, thus, have a positive impact on the development of new therapeutic strategies for reversing or curing prediabetes and T2D in the future.

## Author Contributions

All authors participated directly in writing, reviewing, and editing. All authors have read and agreed to the published version of the manuscript.

## Funding

This study was supported by grants from the National Institutes of Health of Diabetes and Digestive Kidney Diseases (DK067912, DK112917, and DK102233) to DT, and a postdoctoral fellowship from the Larry L. Hillblom Foundation (#2020-D-018-FEL) to JH.

## Conflict of Interest

The authors declare that the research was conducted in the absence of any commercial or financial relationships that could be construed as a potential conflict of interest.

## Publisher’s Note

All claims expressed in this article are solely those of the authors and do not necessarily represent those of their affiliated organizations, or those of the publisher, the editors and the reviewers. Any product that may be evaluated in this article, or claim that may be made by its manufacturer, is not guaranteed or endorsed by the publisher.
